# Supplementary material for: Decoding phantom limb movements from intraneural recordings
Source: Nat Commun. 2026 Feb 8;17:2511. doi: 10.1038/s41467-026-69297-0 (PMC12996621; doi:10.1038/s41467-026-69297-0)

**SUPPLEMENTARY MATERIALS**

Decoding phantom limb movements from intraneural recordings

Cecilia Rossi^1^, Marko Bumbasirevic^2^, Paul Čvančara^3^, Thomas Stieglitz^3^, Stanisa Raspopovic^4,5^, Elisa Donati^1,†,^* and Giacomo Valle^6,†,^*

^1^ Institute of Neuroinformatics, University of Zurich and ETH Zurich, Zurich, Switzerland.

^2^ Orthopaedic Surgery Department, School of Medicine, University of Belgrade, Belgrade, Serbia.

^3^ Department of Microsystems Engineering – IMTEK, IMBIT // NeuroProbes, BrainLinks-BrainTools Center, Bernstein Center Freiburg, University of Freiburg, Freiburg, Germany.

^4^ Department of Health Sciences and Technology, Institute for Robotics and Intelligent Systems, ETH Zürich, Zürich, Switzerland.

^5^ Center for Medical Physics and Biomedical Engineering, Medical University of Vienna, Vienna, Austria.

^6^ Department of Electrical Engineering, Chalmers University of Technology, Gothenburg, Sweden.

*Corresponding authors: [elisa@ini.uzh.ch](mailto:elisa@ini.uzh.ch); [valleg@chalmers.se](mailto:valleg@chalmers.se)

†Equal contribution

Keywords: intraneural recording, neural interface, phantoms movements, peripheral nervous system, amputees, prosthetics, neuromorphic, Spiking Neural Networks.

**List of Supplementary Materials:**

Fig. S1. Surgical implantation of the intraneural electrodes in the distal branch of the sciatic nerve.

Fig. S2. Neural response during phantom movements in S2.

Fig. S3. Performance of conventional neural decoders using different signal features.

Fig. S4. Motor modulation and sensory restoration in S2.

Fig. S5. Phantom movements considered during the experimental data acquisition.

Fig. S6. Decoder performance for individual and multiple TIMEs.

Table S1. Participants’ demographics.

Table S2. Muscular innervation of the distal branch of the sciatic nerve.


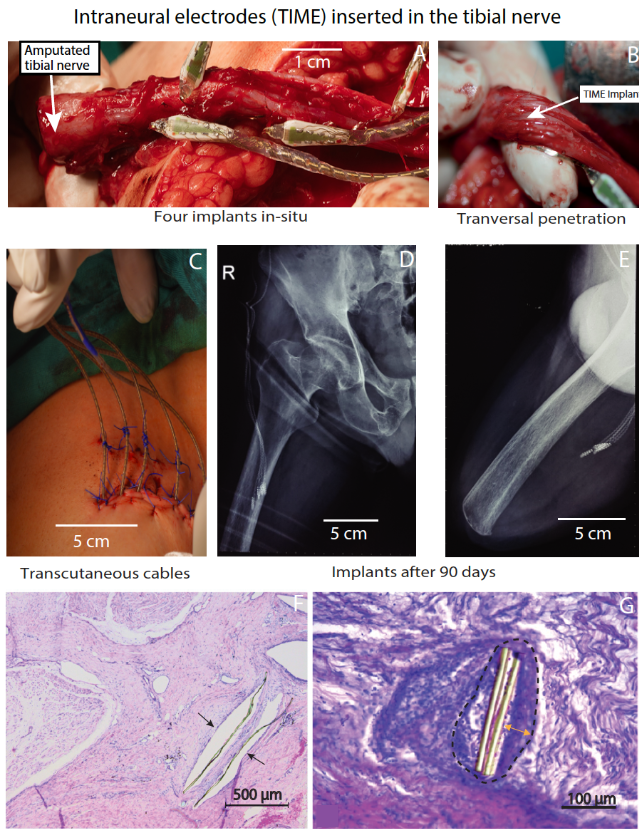


**Fig S1. Surgical implantation of the intraneural electrodes in the distal branch of the sciatic nerve.** A-B) The electrodes are positioned to cross tranverally the amputated tibial branch of the sciatic nerve. C) The electrode cables are tunneled through the thigh and pulled out of the leg through small incisions just a few centimeters below the iliac crest, to enable transcutaneous connection with the neurorecorder. D-E) The placement of the implants within the thigh is shown in the X-ray pictures. Adapted from Petrini et al.^21^. F-G) Left: Hematoxylin and Eosin (HE) staining of implanted nerve sections in S2 is displayed. Arrows indicate the electrode. Right: Photomicrographs of HE stained section of the tibial nerve with an electrode implanted at 20x magnification showing the electrode surrounded by a connective tissue capsule and aggregates of macrophages. Fibrotic encapsulation is indicated within the dashed area (after 90 days). Images taken with permission from Valle et al.^63^.


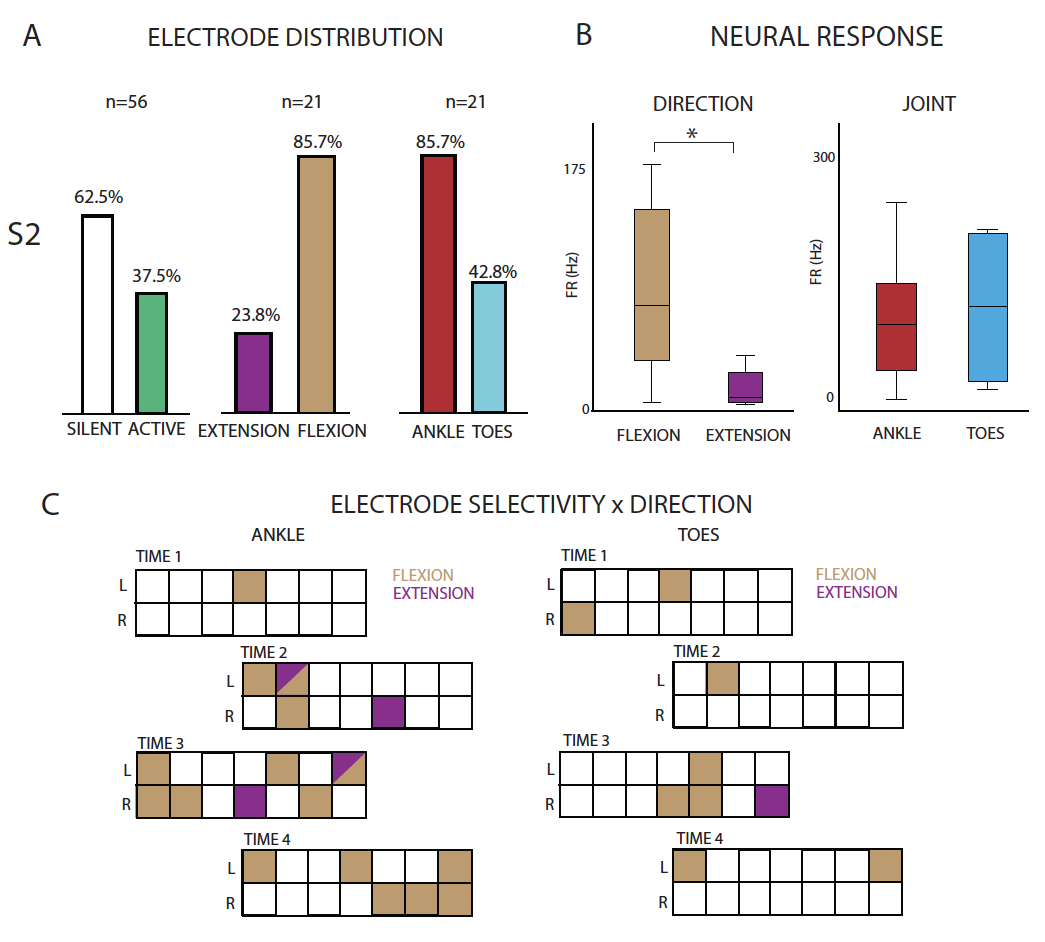


**Fig S2. Neural response during phantom movements in S2.** A) Percentage of significant modulation among all 56 channels. Data for silent vs active, flexion vs extension and knee vs ankle vs toes are reported. n indicates the sample size. B) Average firing rates across all channels for each direction and joint involved. On each box, the center line represents the median, the bottom and top edges of the box represent the 25^th^ and 75^th^ percentiles, respectively. The whiskers extend to the minimum and maximum values within 1.5×IQR from the quartiles. Points beyond the whiskers are shown as outliers. Friedman test *p<0.05 (flex-ext: p=0.00002. ankle-toes: p =0.275). n=56. C) Each of the 4 TIME are reported for the 3 joint movements, showing the significant modulation for each individual channel. A channel colored in purple shows only significant modulation for flexion, in brown only for extension, both colors both directions and white no modulation. Data from S2.


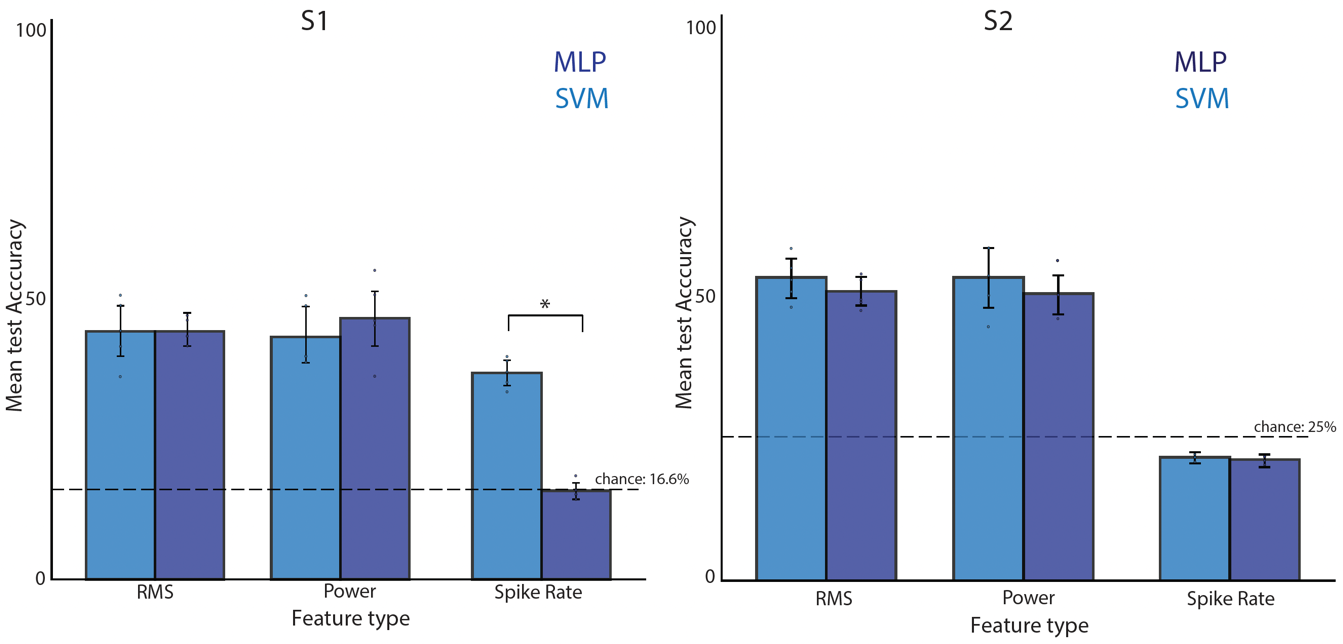


**Fig S3. Performance of conventional neural decoders using different signal features.** Comparison of accuracy of MLP and SVM decoders across various feature types commonly extracted from neural signals, including time-domain (e.g., RMS), frequency-domain (e.g., power spectral density), and spiking-based features. Results of S1 and S2 highlight how feature selection impacts the overall performance of both decoders, with some features yielding significantly higher accuracy than others across subjects. Error bars represent standard deviation across trials. Chance levels are reported with dashed lines for both participants (S1: 16.6%; S2: 25%). Two-sided Paired-t-tests *p<0.0001. n=540 for S1 and n=720 for S2 (5-folds cross validation).


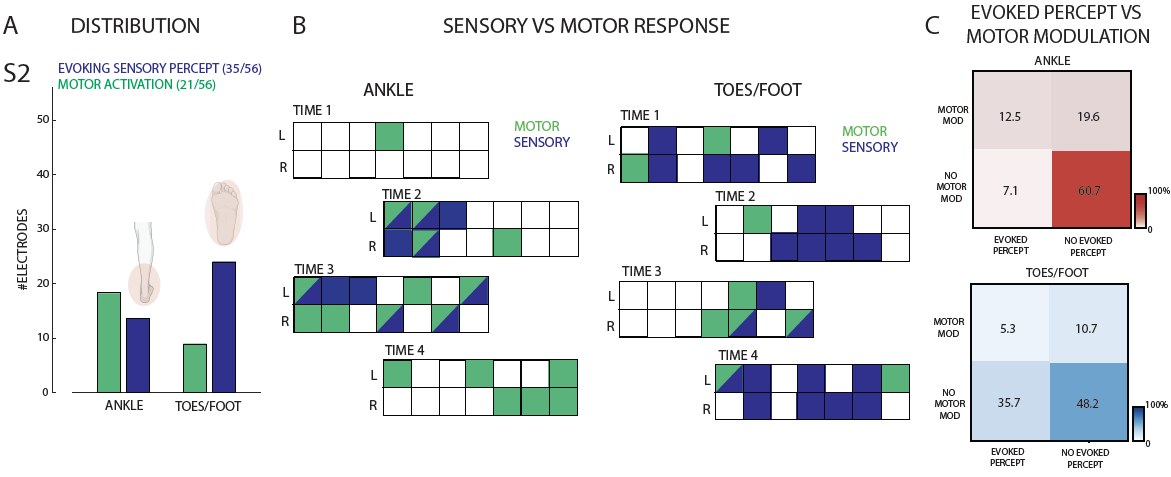


**Fig S4. Motor modulation and sensory restoration in S2.** A) Distribution of the electrodes showing significant motor modulation compared to those evoking sensation in the ankle or foot areas. Icons created in BioRender. Valle, G. (2026) <https://BioRender.com/lxrugya>. B) Each of the 4 TIME are reported for the 2 joints, showing the significant motor modulation (blue) or sensory evoked response (green) for each individual channel. A channel colored in blue shows only significant motor modulation, in green only for evoked-sensation, both colors both responses and white no response. C) Modulation matrices showing the percentage of electrodes significantly modulating only during movement, evoking only sensations or both for the two joints. N=56. Data from S2.


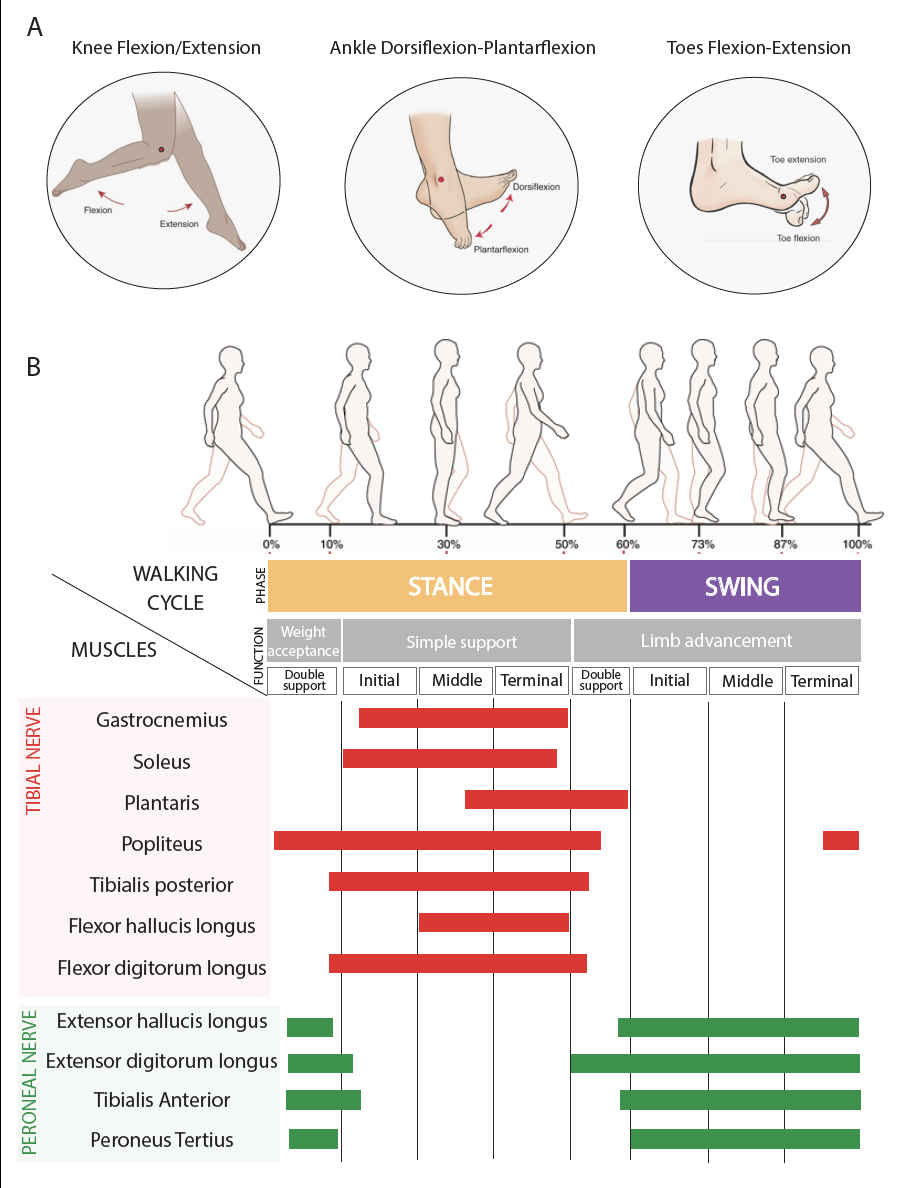


**Fig S5. Types of phantom movements and its muscular representation during gait**. A) Flexion and Extension of the knee, Dorsiflexion and Extension of the Ankle. According to common policy, in this study we refer to the dorsiflexion as extension of the ankle, while we use the term flexion of the ankle to describe the plantarflexion. Toes Flexion and Extension. B) Representation of muscle activation during a gait cycle. The color bars indicate periods where the muscles are active during the gait cycle. Phase, function and nerve innervation are displayed.


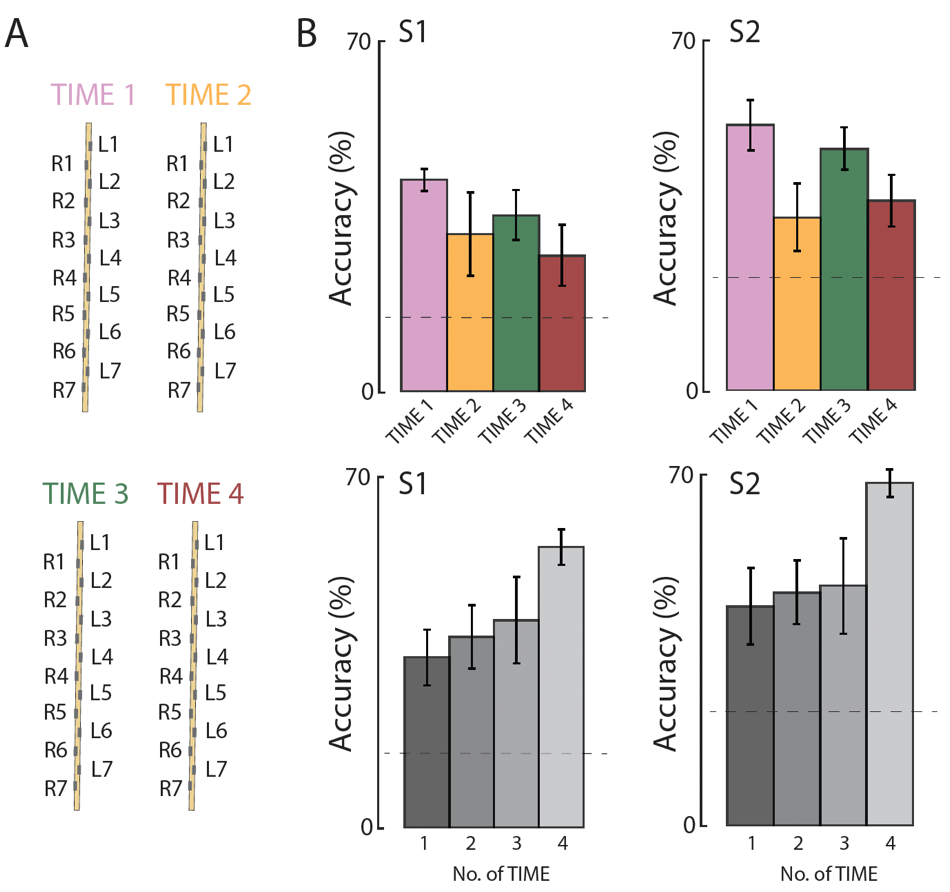


**Fig S6. Decoder performance for individual and multiple TIMEs.** A) Schematic of the 4 TIME electrodes and relative active sites. B) Top: Spiking Neural Network decoder performance on individual TIMEs, averaged across 5 folds. n=14. Bottom: Decoder Performance according to increasing number of TIMEs. Averaged mean test accuracy (on 5-fold cross validation) on the 4 TIME electrodes n=56 (for the single electrode performance, n=14), or different combinations of TIMEs (for 2 and 3 electrode performance, n=28-42).

**Table S1.** **Participants’ demographics.**

**
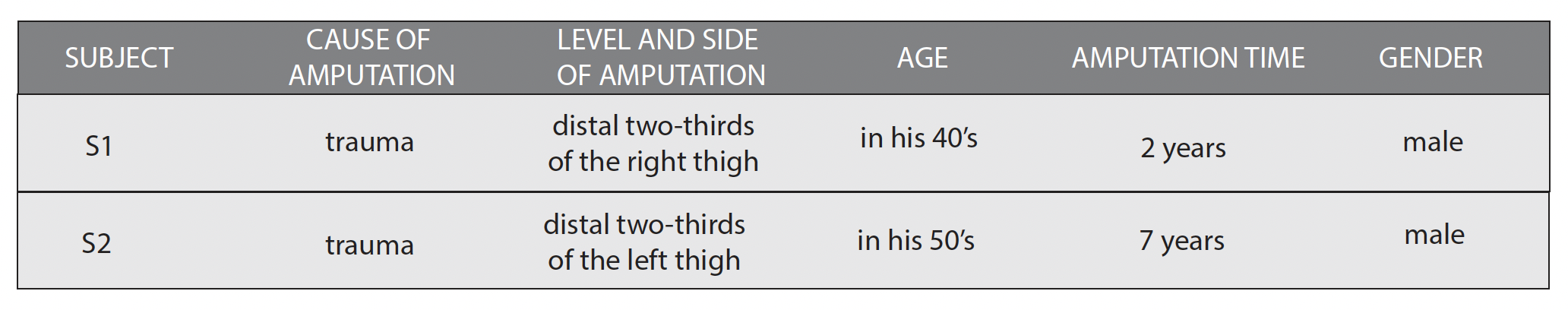
**

**Table S2. Muscular innervation of the distal branch of the sciatic nerve.** In this table we only included the muscles involved in the movements performed in the task, below the amputation level. The muscles innervated by the deep peroneal branch of the sciatic nerve are indicated in green, while the muscles innervated by the tibial branch of the sciatic nerve are indicated in red^36,37^.


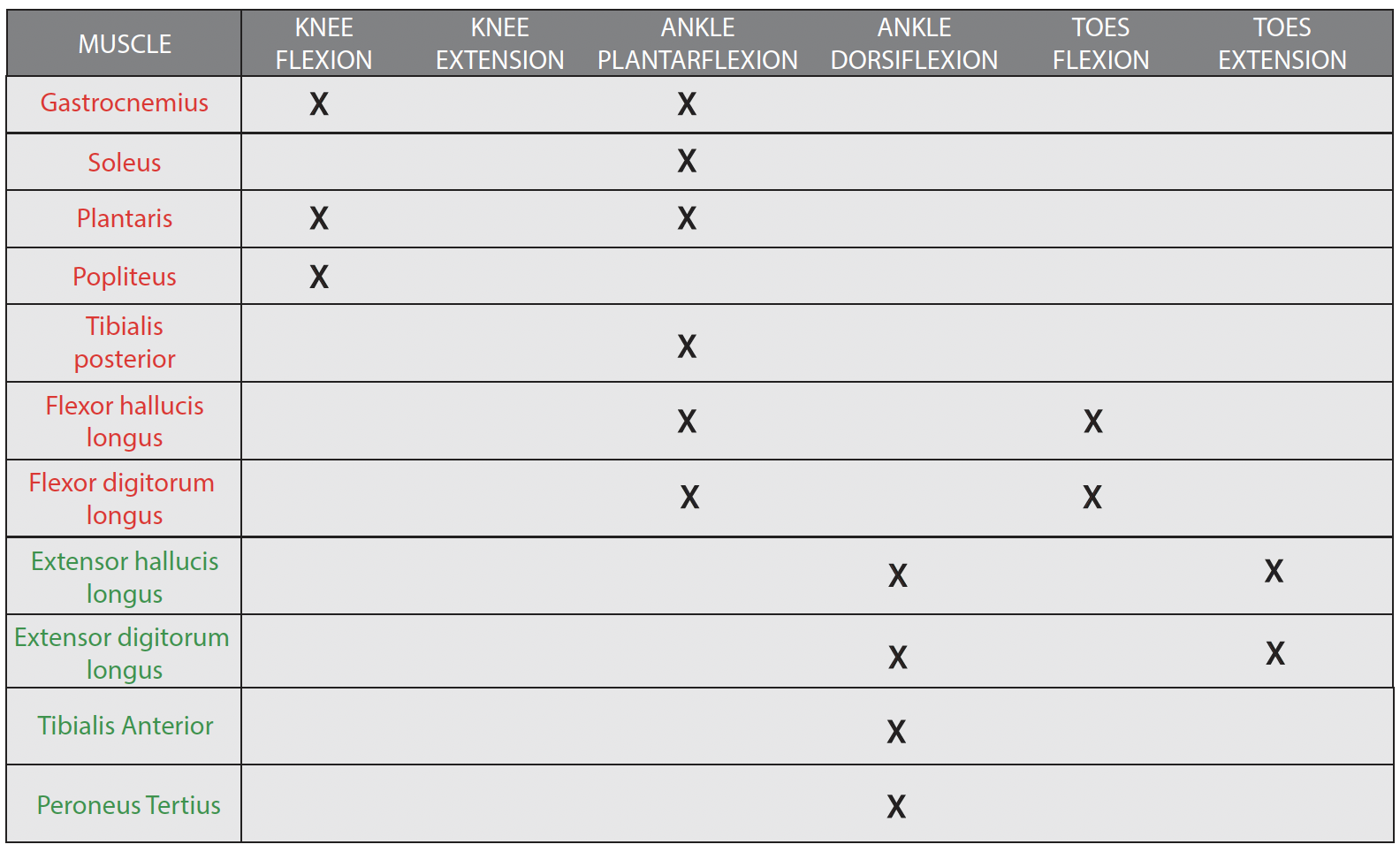

Supplement: Supplementary file 1 — Supplementary Information [file 41467_2026_69297_MOESM1_ESM.docx]
